# Current landscape and challenges of neuro-oncological molecular tumor boards in Germany: results of a nationwide follow-up survey

**DOI:** 10.1007/s11060-026-05549-9

**Published:** 2026-03-31

**Authors:** Lisa S. Hönikl, Sebastian Lange, Bernhard Meyer, Stephanie E. Combs, Alisa M. Lörsch, Anna Lena Illert, Arthur Wagner, Friederike Schmidt-Graf

**Affiliations:** 1 Department of Neurosurgery, Technical University Munich, School of Medicine and Health, Ismaninger Str. 22, 81675 Munich, Germany; 2 Center for Personalized Medicine (ZPM), Technical University Munich, School of Medicine and Health, Munich, Germany; 3 Department of Medicine II, Technical University Munich, School of Medicine and Health, Munich, Germany; 4 Department of Radiation Oncology, Technical University Munich, School of Medicine and Health, Munich, Germany; 5 Department of Medicine III, Faculty of Medicine, Technical University Munich, School of Medicine and Health, Munich, Germany; 6 Department of Neurology, Technical University Munich, School of Medicine and Health, Munich, Germany; 7 Department of Neurology, Klinikum Bogenhausen, Munich, Germany

**Keywords:** Molecular tumor boards, Neuro-oncology, Survey, Targeted therapy

## Abstract

**Background:**

In 2022, a nationwide survey revealed heterogeneity and limited standardization in the implementation of molecular tumor boards (MTBs) for neuro-oncology in Germany. This follow-up study aimed to assess recent developments, structural changes, and the integration of digital tools.

**Methods:**

An anonymous online questionnaire was distributed to approximately 445 members of the German Neuro-Oncology Working Group (NOA). Items focused on MTB availability, frequency, tumor inclusion, recommendation pathways, implementation, digital infrastructure, and perceived barriers.

**Results:**

35 responses were collected. Respondents were primarily experienced neurosurgeons and neurologists working in certified university centers. Most were based in Bavaria, Baden-Württemberg, North Rhine–Westphalia, and Berlin. The number of in-house MTBs increased, with most boards meeting weekly. A broader range of tumor entities—including lower-grade gliomas—and earlier therapy lines are increasingly discussed. Recommendations were issued in fewer than half of cases, and over 30% of proposed therapies were implemented, though often hindered by lack of reimbursement. Virtual tumor boards (50%) and structured databases (40%) are increasingly used, and around 5% of patients are enrolled into national studies. Follow-up strategies and feedback loops remain inconsistent across centers.

**Conclusions:**

MTBs have become more established in neuro-oncology care, reflecting a growing commitment to precision medicine. However, implementation of recommendations remains limited, often constrained by lack of actionable targets, challenges accessing studies or off-label treatments. To fully realize the potential of MTBs, standardized integration pathways, greater harmonization, broader study offerings, and structured evaluation systems are needed.

## Introduction

Precision oncology has increasingly transformed cancer care by tailoring treatments based on molecular tumor profiles, yet neuro-oncology has lagged behind other fields in translating genomic advances into clinical gains [1]. Molecular Tumor Boards (MTBs) have emerged as an essential strategy to interpret complex genomic data and guide personalized treatment decisions. Indeed, MTBs are now considered integral to high-quality oncology care and are recommended to optimize individualized clinical management [2]. In neuro-oncology, however, the application of precision medicine has been particularly challenging [1]. Primary brain tumors such as gliomas are biologically heterogeneous, frequently lack readily “druggable” oncogenic drivers, and often show poor response to systemic therapies. Consequently, standard treatments for gliomas have remained largely limited to surgery, radiotherapy, and alkylating chemotherapy agents such as temozolomide.

In 2022, we conducted the first nationwide survey of neuro-oncological MTBs in Germany, published in BMC Cancer [3]. It revealed broad implementation of MTBs across university centers, but also significant heterogeneity regarding structure, tumor inclusion, recommendation practices, and implementation pathways. Key barriers included lack of reimbursement for off-label therapies, insufficient standardization, and missing follow-up mechanisms. Despite these challenges, respondents recognized the potential of MTBs in advancing individualized care.

Since then, several notable developments have shaped the landscape of neuro-oncology precision medicine. The Phase III INDIGO trial demonstrated a progression-free survival benefit of Vorasidenib, an IDH1/2 inhibitor, in patients with IDH-mutant low-grade gliomas [4]. This marked a milestone for targeted therapy in CNS tumors and reinforced the relevance of molecular profiling. In parallel, the European Association of Neuro-Oncology (EANO) issued 2023 guidelines on molecular testing in gliomas, defining clinically relevant biomarkers and recommending structured testing algorithms [5]. More recently, the European Society for Medical Oncology (ESMO) proposed consensus-based quality indicators for MTBs, including standardized reporting, follow-up procedures, and performance metrics [6]. Such frameworks from ESMO and others aim to standardize MTB practice internationally, thereby accelerating the integration of precision medicine into routine oncology care. Notably, Germany has also launched a national Network for Personalized Medicine to enhance patient care and research translation, reflecting a broader commitment to collaborative precision oncology infrastructure [1].

Despite these progresses, many challenges persist: limited availability of effective targeted therapies for brain tumors, restricted trial access, inconsistent insurance coverage, and variable integration of MTB decisions into clinical workflows.

To assess how these developments have influenced practice, we conducted a follow-up survey in 2025 among German neuro-oncology centers. Our aim was to evaluate changes since 2022, identify persistent gaps, and explore the adoption of digital tools and structural innovations. This study provides an updated national overview of MTB integration in neuro-oncology and informs future strategies for harmonizing precision medicine across institutions.

## Methods

### Survey design and distribution

A descriptive, anonymous online survey was conducted among members of the Neuro-Oncology Working Group (NOA) of the German Cancer Society. The questionnaire was distributed electronically via email, and participation was open between August 1 and October 31, 2025 (13 weeks). All NOA members (~445 individuals at the time of the survey) were invited to participate voluntarily without incentives. The survey was administered using the secure online platform MySurvio (Survio s.r.o., Brno, Czech Republic).

The survey was intentionally designed as an anonymous, cross-sectional assessment to encourage open participation and protect respondent confidentiality. As a result, matching individual responses or centers across timepoints (2022 and 2025) was not possible, and the longitudinal comparison is therefore descriptive in nature.

### Questionnaire development

The survey was based on the instrument used in the initial 2022 assessment, with modifications to capture recent developments in neuro-oncology and precision oncology workflows. It consisted of 34 structured questions covering: Professional background and institutional setting, MTB availability, frequency, and organizational format, tumor types and clinical situations discussed in MTBs, disciplines involved in MTB meetings, recommendation rates and implementation frequency, barriers to therapy access, including reimbursement, use of digital tools (e.g., structured databases, virtual tumor boards, AI support) and perceived therapeutic success of targeted therapies.

Questions were presented in single-choice or multiple-choice format. No free-text responses were included in this version.

## Results

### Participant demographics and institutional context

A total of 35 fully completed questionnaires were returned, corresponding to a response rate of approximately 8% based on the 445 members of the German Neuro-Oncology Working Group (NOA) invited to participate. Of these 35 fully completed responses, the majority of participants were specialists in neurosurgery (46%) or neurology (38%), accounting for over 80% of respondents. Radiation oncologists, neuropathologists, and medical oncologists each comprised 6% of responses, while no neuroradiologists participated. Most respondents (91%) were attending-level physicians, including 60% as fully certified specialists and 31% with additional qualifications in medical tumor therapy. Only 9% were residents or fellows. More than 90% reported having over five years of neuro-oncology experience, with 71% stating over ten years.

Institutionally, 74% of participants worked at university hospitals, 20% at affiliated teaching hospitals, and only 6% in outpatient specialty centers. Notably, 91% reported working in DKG-certified neuro-oncology centers, an increase from 71% in 2022. Regionally, responses covered nine German federal states, with highest representation from Bavaria (23%), Baden-Württemberg (20%), and North Rhine–Westphalia (20%). No responses were received from Hamburg, Bremen, Saarland, or Brandenburg.

### Availability and structure of MTBs

Access to MTBs was nearly universal: 74% had an in-house MTB, and 20% participated through referral. Only 6% reported no access. Notably, 20% of respondents indicated that they do not host an in-house MTB but regularly refer neuro-oncology patients to an external center for molecular case review. Additionally, 46% of respondents reported having standardized referral systems (e.g., SOPs) in place for assigning patients to MTBs, while 29% indicated only partial structures and 26% reported no such system. Regarding access for smaller or non-university hospitals, 54% described it as unproblematic, 31% noted that it depends on individual case decisions, while 6% each reported either significant bureaucratic barriers or no access at all.

Meeting frequencies were predominantly weekly (63%), followed by biweekly (20%) and monthly (17%).

Regarding format, 57% discussed neuro-oncological cases in general MTBs covering all tumor types, 31% used organ-specific tumor boards with molecular components, and 11% held dedicated neuro-oncology MTBs. Compared to 2022, there was an increase in centralized, entity-independent MTB structures.

### Patient selection and tumor entity inclusion

Neuro-oncology patients were included regularly in MTB discussions by 71% of respondents, rarely by 23%, and not at all by 6%. High-grade tumors such as glioblastoma (83%), diffuse midline glioma (89%), and IDH-mutant grade 4 astrocytoma (83%) were most frequently discussed. Inclusion of grade 2 gliomas and grade 2 meningiomas increased modestly compared to 2022, indicating a broader approach to molecular profiling.

See Table 1 for a comparison of tumor entities discussed in 2022 vs. 2025.

**Table 1:** Neuro-oncological tumor entities included in MTBs − 2022 vs 2025

Tumor entity (WHO 2021)	2022(%)	2025(%)
Glioblastoma, IDH-wildtype (Grade 4)	79%	83%
Diffuse midline glioma, H3 K27-altered (Grade 4)	74%	89%
Astrocytoma, IDH-mutant (Grade 4)	68%	83%
Astrocytoma, IDH-mutant (Grade 3)	61%	63%
Astrocytoma, IDH-mutant (Grade 2)	42%	49%
Oligodendroglioma, 1p/19q-codel (Grade 3)	55%	60%
Oligodendroglioma, 1p/19q-codel (Grade 2)	40%	46%
Ependymoma (Grade 3)	55%	63%
Ependymoma (Grade 2)	37%	49%
Meningioma (Grade 3)	55%	69%
Meningioma (Grade 2)	37%	43%
Chordoma	42%	34%
Other (glioneuronal tumors, etc.)	18%	14%

Values indicate the percentage of respondents who reported including each tumor type in MTB discussions (multiple answers possible). WHO CNS grading per 2021 classification

### Timing of case discussion

MTBs were predominantly used at recurrence. 80% of respondents included patients with no remaining standard options, and 74% also included patients at recurrence with remaining options. Presentation at primary diagnosis was less common (46% without standard options, 20% with standard options).

### Multidisciplinary team composition

MTBs commonly included neuropathologists/molecular pathologists (82.9%) and medical oncologists (83%). Neurologists participated in 69%, neurosurgeons in 49%, and radiation oncologists in 37%. Neuroradiologists were present in 31%. Molecular coordinators or report authors (“Befundersteller”) were involved in 57%, while only 9% mentioned additional roles such as bioinformaticians.

### Case annotation and decision-making

Annotation responsibilities varied: 37% of respondents cited MTB members from other specialties (e.g., molecular pathology), while 23% had a designated full-time annotator. Neurosurgeons (43%) and neurologists (29%) also frequently prepared cases. Only 3% assigned annotation to residents.

### Recommendation and implementation rates

**Fig. 1:**
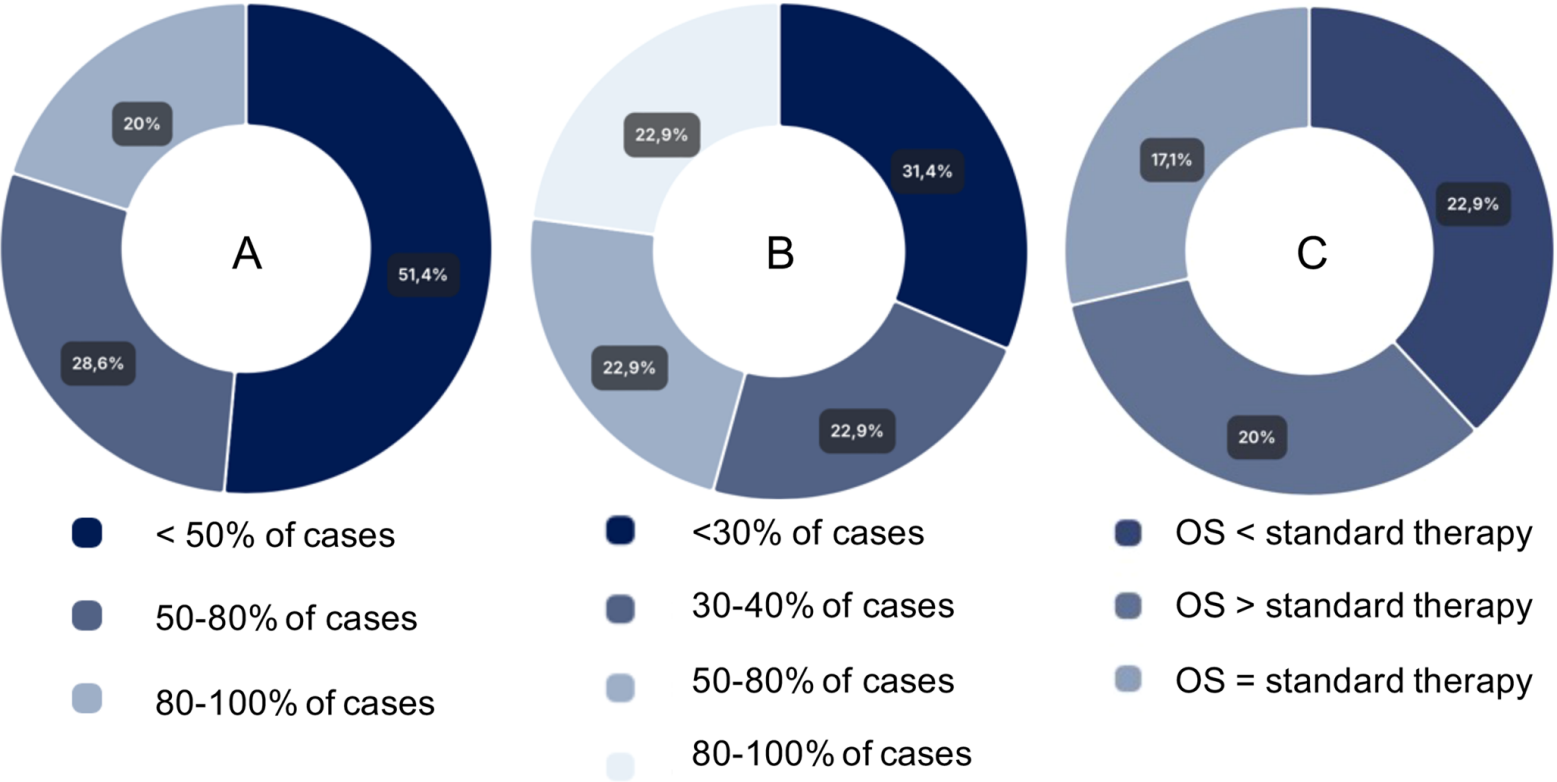
Responses from neuro-oncology experts (*n* = 35 complete survey participants). Values represent the percentage of respondents selecting each category. (**A**) estimated frequency of cases in which an MTB issues a therapy recommendation. (**B**) estimated frequency of cases in which recommended therapies are actually implemented. (**C**) subjective clinical impression of overall survival (OS) in patients receiving MTB-recommended therapies, compared to standard therapy

Only 20% of respondents reported that recommendations were issued in 80–100% of MTB cases; over half (51%) stated that < 50% of cases received a recommendation (Fig. 1A). Implementation was even lower: just 23% reported implementation in >80% of cases, while 54% implemented < 50% of recommendations (Fig. 1B). These represent declines compared to 2022.

In most cases, the treating physician within the neuro-oncology team—typically a neurologist (60%) or oncologist (53%)—is responsible for implementing the recommended targeted therapy.

When asked about the clinical response observed after implementing MTB-recommended therapies, 37% of respondents reported a clinical benefit in at least some patients, such as disease stabilization or partial response (Fig. 1C). These assessments reflect subjective physician impressions, not based on formal analyses within their respective institutions based on standardized clinical outcome measures such as progression-free survival (PFS), overall survival (OS), or objective response rate (ORR). However, 26% reported that clinical response was rare or absent and the therapy was poorly tolerated, 23% indicate that it has so far shown limited efficacy and performs worse than standard therapy, 3% report that no experimental therapy has been administrated to date and 12% state that they are unable to provide an assessment (Fig. 1C). These findings highlight the heterogeneous and often limited real-world effectiveness of targeted therapies in neuro-oncology, despite molecular rationale.

### Barriers to implementation

Major barriers included lack of reimbursement for off-label treatments and patient deterioration before therapy initiation. 71% cited difficulties with reimbursement under statutory insurance. Encouragingly, only 9% reported that > 50% of patients died before therapy; 74% indicated this occurred in <30% of cases.

Additional data revealed that 43% of respondents did not experience routine cost coverage issues for molecular diagnostics, while 31% encountered such problems primarily with patients under public insurance. A smaller proportion (9%) addressed costs via inclusion under the §64e research funding framework in Germany, while 17% reported not using §64e.

### Evidence thresholds for recommendations

Recommendations were most often based on pathogenic (60%) or likely pathogenic (46%) variants. Only 9% considered variants of unknown significance. Regarding evidence levels, 31% required high-level clinical evidence, 43% considered preclinical data in selected cases, and 26% had no defined threshold. Roughly one-third of respondents reported using formal evidence frameworks such as ESCAT or the EANO classification to support clinical decision-making.

### Use of digital tools

Digital tools were used: 50% used virtual MTBs, 40% structured databases. No respondents reported use of AI-based annotation tools. A total of 46% of respondents reported that outcome tracking after MTB discussions is performed systematically; 37% indicated that follow-up is conducted partially or on an ad hoc basis, while 17% stated that no structured follow-up is in place.

In terms of future potential, 69% of respondents supported nationwide implementation of structured databases, 37% anticipated that virtual tumor boards would become standard, and 29% saw a potential role for large language models (e.g., ChatGPT) in assisting variant interpretation. These responses indicate growing expectations for digital and AI-supported tools in molecular decision-making.

### Trial access

Enrollment of neuro-oncology patients in the nationwide NCT/DKTK MASTER program remained limited. The NCT/DKTK MASTER (National Center for Tumor Diseases/German Cancer Consortium – Molecularly Aided Stratification for Tumor Eradication Research) program is a nationwide German initiative that provides comprehensive molecular tumor profiling for patients with rare or advanced cancers, aiming to identify personalized treatment options and support decision-making in molecular tumor boards. While 43% of responders reported occasional enrollment (ranging from 1 - 4 patients per year), 57% did not enroll any patients into MASTER, mirroring previous survey results. When asked about trial access through MTBs, 51% of respondents estimated that approximately 5% of patients could be enrolled in interventional or registry studies. However, 29% reported no patient enrollment at all, and only 20% of respondents reported inclusion rates above 5%, underscoring limited real-world study access for most patients discussed in MTBs.

## Discussion

This follow-up survey provides an updated panorama of how Molecular Tumor Boards are utilized in neuro-oncology across Germany, revealing both encouraging developments and persistent challenges since the initial 2022 survey. Given the low response rate, the findings primarily reflect insights from highly specialized neuro-oncology centers with established MTB structures. Overall, our findings indicate that MTBs have become more entrenched in neuro-oncological practice – they are more widely available, integrated somewhat earlier in the disease course, and supported by emerging digital infrastructure – yet the impact of MTBs on patient outcomes remains limited by systemic barriers, particularly the difficulty of delivering off-label targeted therapies. In this discussion, we contextualize key changes over time, examine regional and structural disparities, evaluate the adoption of new tools, and discuss the implications of recent advances (WHO classification, targeted drugs, guidelines) on MTB practice. We also propose considerations for improving MTB effectiveness and standardization moving forward.

### Expanded adoption and institutionalization of MTBs

Compared to 2022, a greater proportion of neuro-oncology providers now report having a dedicated in-house MTB [3]. This suggests that some centers which previously relied on referring cases to external MTBs have established their own, likely to streamline workflow and focus on their patient population. Our data (74% in-house in 2025 vs 66% in 2022) align with anecdotal reports of several German university clinics launching precision oncology programs in the interim [3]. Notably, no respondents in 2022 said they lacked MTB access, whereas 6% did in 2025 – this small difference might simply reflect that in 2022 non-MTB centers chose not to respond, whereas in 2025 a few did [3]. Nonetheless, effectively all major neuro-oncology centers have MTB access now, which represents a maturation of the field. This mirrors trends internationally, as precision medicine becomes a standard of care in oncology; for example, by 2023 most comprehensive cancer centers globally have some MTB program. The fact that > 90% of our respondents work in certified centers and >94% have MTB access suggests that in Germany, neuro-oncology MTBs are now a de facto feature of advanced care – a significant change from five years ago when such boards were nascent or absent in many places.

### Frequency and workflow adjustments

Weekly MTB meetings remain standard, reflecting that centers accumulate enough cases (or multi-tumor boards cover enough volume) to justify weekly reviews. There was a modest shift from monthly to biweekly schedules for some, indicating that even mid-sized centers might handle cases more frequently now, possibly due to increased sequencing of recurrent tumors. One of the most striking changes was in the format of MTB discussions: a clear move toward entity-agnostic MTBs. In 2022 [3], half of respondents discussed neuro cases in their organ-specific tumor board, effectively treating MTB as an adjunct to the regular neuro-oncology board. By 2025, this dropped to ~31%. Concurrently, the use of a unified molecular board for all tumor entities rose to ~57%. This suggests that many institutions realized efficiency or quality benefits in consolidating expertise – a molecular board that convenes diverse experts (genomic oncologists, pathologists, etc.) can handle cases from any specialty, including neuro-oncology, often yielding deeper molecular insight than an organ-specific team alone. It may also reflect workforce constraints: having separate MTBs for every specialty is resource-intensive, so combining them is pragmatic. On the other hand, a few centers did spin off dedicated neuro-MTB meetings (11%, up from 5%), likely those with very high neuro-oncology case volumes or specialized research programs (e.g. a neuro-focused precision trial portfolio). These centers perhaps found that neuro-oncology has unique considerations (blood-brain barrier, neuropathology nuances, etc.) warranting a separate forum. Both approaches have merit, and the optimal model may depend on institutional size. ESMO’s recent recommendations do not mandate organ-specific MTBs [6], but emphasize having all needed expertise available. Many German centers appear to be following the model of a central MTB with ad hoc disease-specific input, which can work well as long as neuro-oncology representatives are present and follow-up back to the disease team is ensured. The integration of MTB with standard care was an area of inconsistency flagged in 2022; our results show some progress in clarity – most have chosen a defined pathway (general vs specific), reducing ad-hoc approaches.

### Regional disparities

While MTBs are broadly available across Germany, our 2025 survey reveals uneven regional participation. Most responses came from large western and southern states (e.g., Bavaria, Baden-Württemberg, NRW) and Berlin, while no responses from Hamburg, Brandenburg, or Saarland suggest lower engagement or possible gaps in local MTB availability. Still, the proportion of centers relying on external MTBs has decreased (from 34% to 20%), indicating growing local capacity. Encouragingly, 66% of respondents reported using virtual platforms, which may help bridge geographic gaps and support broader access to molecular case discussions.

### Multidisciplinarity and clinical expertise

MTBs remain highly multidisciplinary, with consistent participation from neuropathologists and a growing presence of medical oncologists (83%). This shift may reflect increasing involvement of precision oncology programs and enhanced systemic therapy expertise. Decreased attendance from radiation oncologists and neurosurgeons likely reflects case composition and evolving clinical needs. Notably, approximately one-third of respondents were unfamiliar with variant classification thresholds, underscoring the need for continuous genomics education and harmonized evidence frameworks, as emphasized by EANO and ESMO guidelines.

### Earlier patient inclusion and case selection

A positive development since 2022 is the earlier inclusion of patients in MTBs—74% of centers now present recurrent cases even when standard options remain [3]. This aligns with current oncology paradigms promoting earlier genomic work-up to prepare for rapid disease progression. Fewer centers now present newly diagnosed patients if standard therapy is available, reflecting a more targeted and resource-conscious MTB approach. These practices are in line with EANO 2023 [5] recommendations, which highlight specific indications for molecular testing at diagnosis (e.g., BRAF mutations in gliomas).

### Targetability and implementation barriers

Despite broader MTB integration, the proportion of actionable recommendations remains limited: over half of centers report issuing recommendations in less than 50% of cases. Even more concerning, only 23% implement > 80% of their recommendations—down from 47% in 2022. The main barrier remains reimbursement: 71% reported difficulties obtaining approval for off-label targeted therapies in publicly insured patients. Although coverage for molecular diagnostics improved slightly (43% reported no issues), the translation of MTB findings into clinical action remains constrained by systemic factors. Encouragingly, fewer patients now deteriorate before treatment can be initiated, suggesting potential benefits from earlier MTB involvement if financial barriers can be addressed.

### Follow-up and outcome tracking

Only 31% of respondents reported systematic follow-up of MTB recommendations, despite increasing emphasis on outcome documentation in recent guidelines. Without such data, assessing clinical benefit and justifying off-label use remains challenging. Initiatives like prospective registries or structured databases—currently adopted by ~40% of centers—could enable better longitudinal tracking and foster outcome-based learning across institutions.

### Digital tools and AI

Digital infrastructure has progressed modestly, with broad use of virtual MTBs and increasing adoption of structured case databases. However, artificial intelligence (AI) tools, including large language models, are not yet integrated into MTB workflows. Although 28.6% of respondents anticipate future relevance, concerns about reliability, regulatory approval, and data privacy may explain the current absence. Future development of validated, domain-specific AI applications could support variant interpretation and trial matching, but at present, human expertise remains central.

### Limitation

Despite the valuable insights gained, the relatively low number of respondents (35 out of approximately 445 NOA members; ~8%) represents a key methodological limitation. The responses predominantly stem from highly specialized neuro-oncology centers—91.4% of participants work in DKG-certified institutions, and 74.3% are based at university hospitals. This suggests a strong selection bias toward academically active sites with established MTB infrastructures. It is therefore likely that the survey overrepresents centers with more advanced implementation of molecular tumor boards, while perspectives from smaller, less resourced hospitals or outpatient settings are underrepresented. However, this pattern may also reflect the current reality: in many institutions, only one or two individuals are closely involved in MTB activities and possess in-depth expertise in molecular diagnostics and precision oncology. Contributing factors may include institutional variability in MTB integration, limited funding or training opportunities, and uncertainty about the clinical utility of precision medicine in neuro-oncology. These limitations highlight the need to interpret the results as reflective of the most engaged segment of the field and underscore the importance of broader outreach and capacity-building initiatives to promote equitable and widespread adoption of MTB practices across all levels of neuro-oncological care.

## Conclusion

In summary, MTBs have become a routine element of neuro-oncology care in Germany, with increased in-house availability, expanded use of virtual and digital tools, and earlier integration into clinical workflows. Yet, the translation of MTB recommendations into patient benefit remains limited by the scarcity of actionable targets, challenges in trial access, and persistent reimbursement barriers—particularly for off-label treatments in publicly insured patients. Harmonized referral pathways, structured outcome tracking, and continued implementation of national and international guidelines (EANO, ESMO) will be essential to improve consistency and quality. The development of national networks and adoption of AI-assisted tools may offer additional support in the future. Addressing these gaps is key to realizing the full potential of precision medicine for patients with CNS tumors.

## Data Availability

The datasets generated during and/or analyzed during the current study are available from the corresponding author on reasonable request.
